# Epigallocatechin-3-Gallate Alleviates Liver Oxidative Damage Caused by Iron Overload in Mice through Inhibiting Ferroptosis

**DOI:** 10.3390/nu15081993

**Published:** 2023-04-21

**Authors:** Chunjing Yang, Aimin Wu, Liqiang Tan, Dandan Tang, Wei Chen, Xin Lai, Ke Gu, Junzhou Chen, Daiwen Chen, Qian Tang

**Affiliations:** 1 College of Horticulture, Sichuan Agricultural University, Chengdu 611130, China; 2 Tea Refining and Innovation Key Laboratory of Sichuan Province, Chengdu 611130, China; 3 Institute of Animal Nutrition, Sichuan Agricultural University, Chengdu 611130, China; 4 Key Laboratory for Animal Disease-Resistance Nutrition of China Ministry of Education, Sichuan Agricultural University, Chengdu 611130, China

**Keywords:** Epigallocatechin-3-Gallate (EGCG), iron metabolism, ferroptosis, antioxidation

## Abstract

Ferroptosis, a form of regulated cell death, has been widely explored as a novel target for the treatment of diseases. The failure of the antioxidant system can induce ferroptosis. Epigallocatechin-3-Gallate (EGCG) is a natural antioxidant in tea; however, whether EGCG can regulate ferroptosis in the treatment of liver oxidative damage, as well as the exact molecular mechanism, is unknown. Here, we discovered that iron overload disturbed iron homeostasis in mice, leading to oxidative stress and damage in the liver by activating ferroptosis. However, EGCG supplementation alleviated the liver oxidative damage caused by iron overload by inhibiting ferroptosis. EGCG addition increased NRF2 and GPX4 expression and elevated antioxidant capacity in iron overload mice. EGCG administration attenuates iron metabolism disorders by upregulating FTH/L expression. Through these two mechanisms, EGCG can effectively inhibit iron overload-induced ferroptosis. Taken together, these findings suggest that EGCG is a potential ferroptosis suppressor, and may be a promising therapeutic agent for iron overload-induced liver disease.

## 1. Introduction

Iron, an essential element for maintaining health in all organisms, is required for many functional cellular processes, such as mitochondrial respiration, DNA synthesis, and oxygen transport during evolution, and is required in enzymes regulating the respiratory complex [1].

In mammals, iron transport, uptake, and storage are tightly regulated by different pathways and proteins to maintain iron homeostasis. Iron insufficiency causes anemia, while iron overload promotes oxidative stress as a result of its redox reactivity [2].

Ferroptosis, first proposed in 2012 by Dixon [3], is a type of cell death mode that is different from apoptosis and autophagy, and is marked by the iron-dependent accumulation of lipid hydroperoxide to lethal levels [4]. This iron-dependent death depends on three important procedures: Fenton catalysis resulting in oxidative stress, which is caused by the accumulation of free iron; the depletion of the antioxidant GSH, which leads to oxidative stress; and lipid oxidative damage of the cell membrane [5]. Spleen dysfunction and hepatotoxicity are the most common pathological symptoms of iron overload. These multiple organ dysfunctions are caused by the release of excess free iron through the breakdown of heme, which is ubiquitous in the reticuloendothelial organs [6]. Reactive oxygen species (ROS) and oxidative stress can be caused by an increase in free iron [7], as well as decreased cellular stores of antioxidants [8]. Numerous genes have been identified to regulate ferroptosis or serve as markers for it [9]. Negative regulators can inhibit ROS and limit cellular iron increase, such as glutathione peroxidase 4 (GPX4) [10], nuclear factor erythroid 2-related factor 2 (NRF2) [11], solute carrier family 7 member 11 (SLC7A11), and solute carrier family 3 member 2 (SLC3A2) [12]. On the contrary, transferrin receptor 1 (TFR1), NADPH oxidase, and P53 have a positive effect on ferroptosis progress [13]. Previous research has revealed that ferroptosis is related to multiple pathological cell death courses, including cancer cell death, neurodegeneration, inflammation, and infection, as well as liver damage and fibrosis [14]. Ferroptosis regulation has become a powerful therapeutic tool. For this reason, the more ferroptosis regulators that are identified, the more corresponding therapies for ferroptosis-related diseased could be tested and utilized in the future.

Epigallocatechin-3-gallate (EGCG), the most abundant and valuable component in tea plants (Camellia sinensis) [15], constitutes 50–75% of catechins and accounts for approximately 40% of the polyphenols group [16]. EGCG has beneficial effects against oxidation, inflammation, cancer, proliferation, and neurological disorders [17,18,19]. Research has proven that these health benefits are due to its ability to chelate iron and scavenge free radicals [20]. EGCG alleviates intestinal inflammation [21] and the symptoms of Parkinson’s disease [22] by reducing lipid peroxidation. Additionally, it functions by collaborating with oxidative stress, iron dysregulation, and inflammation, shielding against neuronal apoptosis, which is mainly mediated by FPN and hepcidin [23]. The antioxidant properties of EGCG are particularly impressive; its ability to eliminate free radicals and chelate metals has been demonstrated in multiple studies [24,25]. Additionally, some studies have shown that EGCG can inhibit the absorption of non-heme form iron from the intestine [26]. Previous research has also revealed that EGCG treatment can reverse thrombocytopenia, anemia, and leucocytosis [27]. In one study, a patient with chronic lymphocytic leukemia achieved complete molecular remission for a long period of time after consuming EGCG extracted from green tea [28]. However, the mechanism by which EGCG regulates iron metabolism and ferroptosis in the body is still unclear.

In the present study, we constructed an iron-overload mice model to investigate the role of EGCG in iron overload-induced liver injury. We found EGCG could alleviate iron overload-indued liver oxidative damage by regulating iron metabolism and inhibiting ferroptosis. Therefore, this work not only confirmed the ability of EGCG to remove lipid peroxides, but also found a natural treatment for the liver damage caused by ferroptosis.

## 2. Materials and Methods

### 2.1. Chemicals and Antibodies

EGCG (purity ≥ 98%) was provided by Teaturn Bio-pharmaceutical Co., Ltd. (Wuxi, China). Anti-TFR1, FPN, FTH/L, NRF2, pNRF2, GPX4, P62, and HO-1 were purchased from Abcam; anti-KEAP1, SLC3A2, and SLC7A11 were purchased from Cell Signaling Technology; and β-actin was purchased from Santa Cruz biotechnology.

### 2.2. Animal Experiment

The animal experimental procedure was approved by the Institutional Animal Care and Use Committee of the Laboratory Animal Center at Sichuan Agricultural University (SICAU-2015-033). Forty 5-week-old C57BL/6J male mice were used for this study. All mice were kept in the mouse room with air-conditioning (temperature of 24 ± 2 °C, 45–55% humidity, and a 12 h light/dark cycle) and had ad libitum access to food and water.

**Figure 1:**
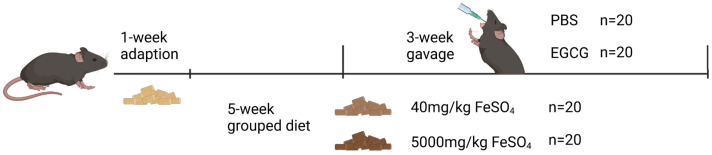
Timeline of the animal experiment. The test mice were divided into four groups as follows. control group: 40 mg/kg FeSO_4_ + PBS; EGCG group: 40 mg/kg FeSO_4_ + 50 mg EGCG/kg body weight; iron group: 5000 mg/kg FeSO_4_ + PBS; iron + EGCG group: 5000 mg/kg FeSO_4_ + 50 mg EGCG/kg body weight. *n* = 10. The picture was created with BioRender.com (accessed on 10 March 2023).

All mice were randomly divided into a 2 × 2 factorial arrangement, fed diets containing 40 mg/kg or 5000 mg/kg FeSO_4_ (the basis of the diet was AIN-93), and gavaged with PBS or 50 mg EGCG/kg body weight per day, respectively. The experiment lasted for 6 weeks, including a 1-week adaptation and a 3-week EGCG gavage; then, all mice were euthanized (Figure 1).

### 2.3. Isolation and Culture of Mouse Primary Hepatocytes

The hepatocytes extracted from euthanized mice from four experimental groups were directly used for flow cytometry analysis, while the in vitro experiments hepatocytes were isolated from the livers of normal C57BL/6J mice. Mouse primary hepatocytes were isolated by digestion using type II collagenase (Gibco™, 17101015). Live hepatocytes were separated via centrifugation using percoll (Sigma-Aldrich, Saint Louis, MO, USA, P1644) and then seeded onto collagen-coated 12-well plates. Primary hepatocytes were cultured in DMEM culture medium for 24 h, followed by washing once with PBS. Fresh DMEM cell culture medium was then added, and cells were treated with different reagents before being harvested at the indicated time points for analysis.

### 2.4. ROS and Lipid ROS Determination

To measure ROS, 50 μM H2DCFDA (Sigma-Aldrich, D6883) was added to primary hepatocytes and incubated for 30 min at 37 °C. Lipid ROS was then detected by staining the cells with 5 μM C11-BODIPY (Thermofisher, Waltham, MA, USA, D3861) for 30 min at 37 °C. After washing the cells three times with PBS, flow cytometry was used to analyze the results.

### 2.5. Measurement of Fe^2+^

To measure the intracellular ferrous iron (Fe^2+^) level, primary hepatocytes were seeded into 12-well plates. When the cells reached about 70% of the plates, they were treated with different concentrations of Fe_3_S_4_ nanozyme (0, 25, 50, 100 μg/mL) for 24 h, and then incubated with 1 μM Far-Red Labile Fe^2+^ dye at 37 °C for 30 min without light. After washing with PBS, the intracellular Fe^2+^ levels were analyzed by flow cytometry.

### 2.6. Blood Parameters Determination

Blood red blood cell count (RBC), hemoglobin (HGB), hematocrit (HCT), mean corpuscular volume (MCV), and mean corpuscular hemoglobin (MCH) were all determined using the automatic biochemical analyzer (Beckman Coulter, München, Germany).

### 2.7. Determination of Iron Parameters

Serum iron, liver, and spleen tissue non-heme iron were determined. The measurements of liver and spleen non-heme iron contents were performed as described previously [12]. Serum iron, total iron binding capacity (TIBC), unsaturated iron binding capacity (UIBC), and serum transferrin (TF) were measured as described previously [29].

### 2.8. Histological Examination of Liver

Histological examination was performed by cutting liver tissue sections from the same part of the lobe, which were then fixed in 4% buffered formalin at 4 °C, embedded in paraffin, and cut into 4 μm-thick sections. HE and Perls staining protocols were applied to the sections, and histopathological changes were observed under a Zeiss Axioscope 5/7 Microscope.

### 2.9. ALT/AST Assay

Serum glutamic oxaloacetic transaminase (AST) and alanine aminotransferase (ALT) were determined by an automatic biochemistry analyzer.

### 2.10. Measurement of Malondialdehyde (MDA) and GSH/GSSG Levels

The level of malondialdehyde (MDA) and glutathione (GSH/GSSG) were assessed using an assay kit from Jiancheng Bioengineering Institute (Nanjing, China), following the manufacturer’s protocol.

### 2.11. qPCR and Western Blot

The extraction of hepatic and splenic total RNA was carried out by TRIzol reagent (Invitrogen, Waltham, MA, USA) according to the manufacturer’s specifications and diluted to 1 μg/uL. The working solution was configured in accordance with the protocols shown in the Reverse Transcription Kit (Takara, San Jose, CA, USA). The ΔΔCT method was used to calculate relative gene expressions by collecting the cycle threshold (Ct) and normalizing it to the housekeeping gene HPRT.

The isolation of liver total protein was performed with a RIPA buffer with phenylmethanesulfonylfluoride (PMSF, 1 mM). Western blotting was carried out as described previously [30]. The primary antibodies were diluted at 1:1000. Secondary goat anti-mouse and anti-rabbit antibodies conjugated with HRP (Santa Cruz, sc-2030 and sc-2031) were diluted to a 1:3000 solution.

### 2.12. Statistical Analysis

This study was carried out as a 2 × 2 two-factorial experiment. The results demonstrated the effects of iron overload and EGCG on mice, and the interaction between high iron and EGCG in mice. All the data are expressed as the means ± standard errors (SEM) using Excel software, and we used the *t*-test and One-way/Two-way Analysis of Variance (ANOVA) to determine the relationships between groups. Statistical significance levels were *p* < 0.05 (*), *p* < 0.01 (**), and *p* < 0.001 (***). All results were generated using GraphPad Prism 9 (GraphPad Software, San Diego, CA, USA).

## 3. Results

### 3.1. EGCG Supplementation Restores Impaired Growth in Mice Caused by Iron Overload

**Figure 2:**
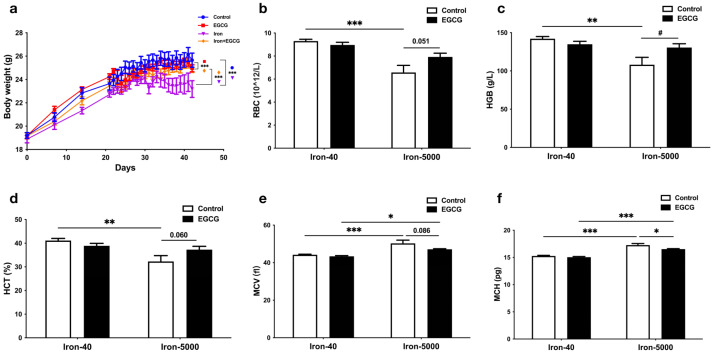
EGCG addition restores impaired growth in mice caused by iron overload. Normal-iron diet (Iron-40) and high-iron diet (Iron-5000) mice were gavaged with EGCG (50 mg/kg body weight per day) or equal volume of PBS separately for 3 weeks, and then serum was collected for analyzing the indexes below. (**a**) The body weight of four groups of mice during the experiment was measured. Mouse body weight was measured weekly for the first three weeks and daily after starting gavage (*n* = 10). (**b**–**f**) The blood RBC (**b**), HGB (**c**), HCT (**d**), MCV (**e**), and MCH (**f**) levels of all the experimental groups were tested after anesthesia and euthanasia of the mice. Data are expressed as means ± SEM. The results of the two-way ANOVA and *t*-test analysis are, respectively, denoted by an asterisk (*) and a hashtag (#), and the remaining results are represented by *p*-values. *p* < 0.05 (*), *p* < 0.01 (**), *p* < 0.001 (***), *p* < 0.05 (#).

To investigate the effect of iron overload in mice and the function of EGCG for mice fed a normal and high-iron diet, we recorded mouse body weight in a time-course manner. The body weight of the mice increased during the adaptation period before the experimental treatment, while a significant difference existed after treatment, with the high-iron group (Iron-5000) being the lowest and a significant recovery occurring after EGCG administration (*p* < 0.001) (Figure 2a).

In addition to causing body weight loss in mice, iron overload led to abnormal blood indices. More specifically, RBC (Figure 2b), HGB (Figure 2c), and HCT (Figure 2d) showed a significant reduction with iron overload (*p* < 0.01), but the concentration of MCV (Figure 2e) and MCH (Figure 2f) increased in iron overload mice (*p* < 0.001), which means anemia occurred in the iron overload groups. Interestingly, EGCG has little regulatory effect on these indices under normal circumstances, but its addition significantly increased HGB (Figure 2c) content in the iron overload condition, and the RBC number (*p* = 0.051, Figure 2b) and HCT level (*p* = 0.0600, Figure 2d) tended to increase.

### 3.2. EGCG Improves Iron Metabolism Disorders Caused by Iron Overload

**Figure 3:**
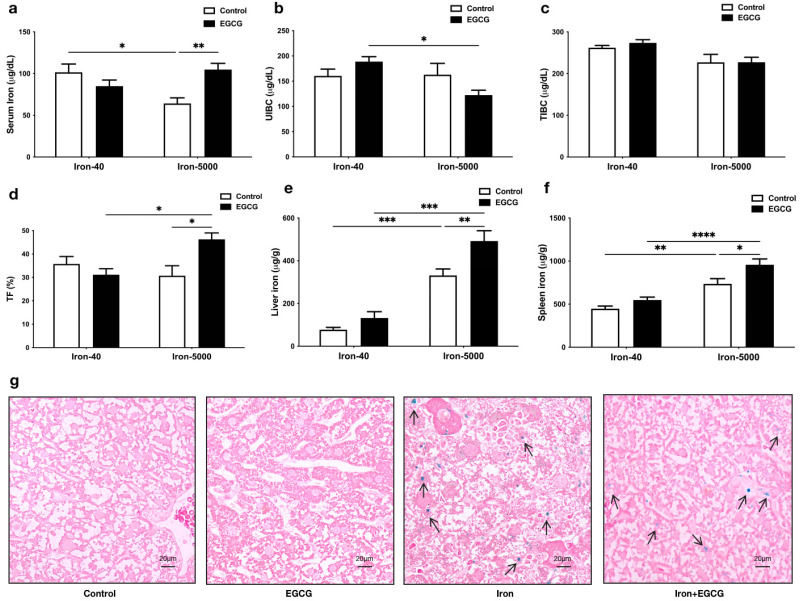
EGCG appears to contribute to the maintenance of iron homeostasis in mice fed an iron-rich diet. (**a**–**d**) Serum iron, UIBC, TIBC, and TF were tested in all groups of mice. (**e**,**f**) Iron content of the liver and spleen (*n* = 10). (**g**) The Perls stained sections of mouse liver tissues (*n* = 5). The arrows point to the iron stained in Perls in the liver. Scale bar, 20 μm. The results of the two-way ANOVA are denoted by *p*-values, *p* < 0.05 (*), *p* < 0.01 (**), *p* < 0.001 (***), *p* < 0.0001 (****).

Subsequently, we detected the changes in iron content in the serum, liver, and spleen, and changes in the state of iron ions in serum. Unexpectedly, the serum iron content in the iron overload group was significantly lower than that in the control group, but EGCG administration significantly increased serum iron content to normal levels (*p* < 0.01) (Figure 3a), and the same pattern was observed in serum transferrin saturation (Figure 3d). Additionally, EGCG addition decreased UIBC in iron overload mice, but it did not affect TIBC content (Figure 3b,c). It is worth noting that excess iron can accumulate in the liver and spleen (*p* < 0.001), and the addition of EGCG allows more iron to accumulate (*p* < 0.01) (Figure 3e,f). The Perls staining results of the liver tissue also demonstrated a similar trend, with the arrows in Figure 3g indicating iron in the liver (Figure 3g). In combination with the blood parameters, we found that EGCG can address anemia caused by iron overload.

### 3.3. EGCG Addition Attenuates Liver Injury

**Figure 4:**
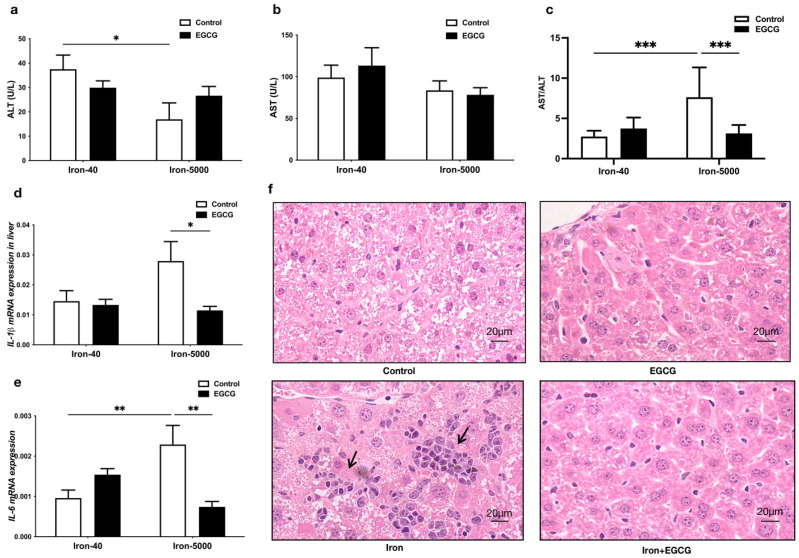
EGCG addition attenuates liver damage and inhibits the mRNA expression of proinflammatory cytokines. (**a**–**c**) Serum ALT, AST, and ALT/AST levels were determined (*n* = 10). (**d**,**e**) The mRNA expression of proinflammatory cytokines *IL-1β* and *IL-6* were tested by qPCR (*n* = 10). (**f**) The HE-stained sections of mouse liver tissues (*n* = 5). The arrows point to areas of inflammatory infiltration in the liver. Scale bar, 20 μm. The results of the two-way ANOVA are denoted by *p*-values, *p* < 0.05 (*), *p* < 0.01 (**), *p* < 0.001 (***).

Figure 4 demonstrates that iron overload significantly increased the AST/ALT ratio in the mouse liver, indicating liver injury. EGCG significantly reduced it (Figure 4c). Furthermore, mRNA levels of the inflammatory factors *IL-1β* and *IL-6* in the liver were also increased by high iron, and EGCG could effectively reduce the levels of these inflammatory factors (Figure 4d,e). However, EGCG had no visible effect on the normal mouse liver. Lastly, histological sections of livers stained with HE revealed that iron overload caused inflammatory infiltration in the mouse liver (indicated by the arrow in Figure 4f), and the addition of EGCG alleviated this inflammatory injury, with liver cells intact and arranged in an orderly manner (Figure 4f). Nevertheless, in the normal mouse liver, EGCG seemed to have no effect.

### 3.4. EGCG Relieves Oxidative Stress and Hepatocytes Ferroptosis Caused by Iron Overload

**Figure 5:**
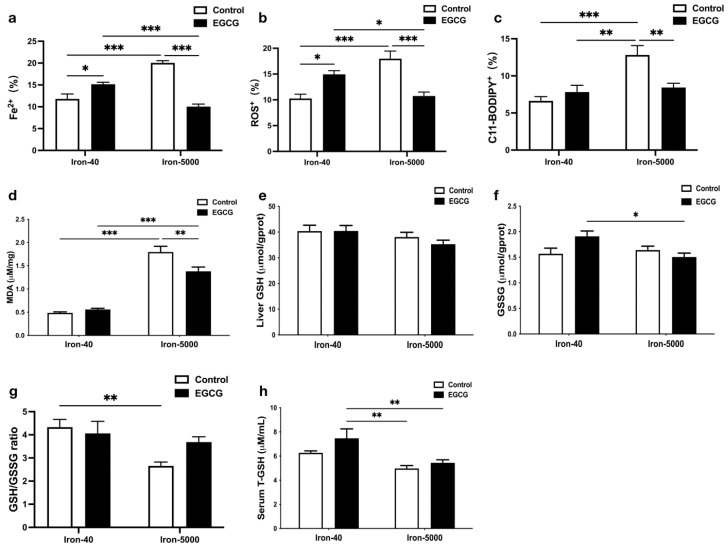
EGEG administration decreases liver oxidation damage in iron overload mice. (**a**–**c**) Level of Fe^2+^, ROS, and C11-BODYPY in the liver (*n* = 8), the percentage (%) unit stands for the area percentage of Fe^2+^, ROS, and C11-BODYPY. (**d**) MDA levels in the liver (*n* = 10). (**e**,**f**) Total GSH and GSSG levels in the liver (*n* = 10). (**g**) Liver GSH/GSSG ratio. (**h**) Total GSH levels in serum (*n* = 10). The results of the two-way ANOVA are denoted by *p*-values, *p* < 0.05 (*), *p* < 0.01 (**), *p* < 0.001 (***).

We tested Fe^2+^, ROS, and C11-BODIPY levels in liver tissues, and they all showed a significant injury to the liver, while EGCG alleviated it (Figure 5a–c). This is also indicated by the determination of in vitro experiments (Supplementary Materials). Generally, iron overload promotes oxidative stress as a result of its redox reactivity [2]. As expected, the MDA content in the iron overload group was markedly higher than that in the control group (*p* < 0.001), but it was decreased after the addition of EGCG compared to the iron overload group (*p* < 0.01) (Figure 5d), indicating EGCG may be a ferroptosis inhibitor. Surprisingly, the effects of excessive iron intake on serum and liver GSH or GSSG did not reach statistical significance (Figure 5e,f,h), while high iron significantly decreased the GSH/GSSG ratio (Figure 5g) (*p* < 0.01), which also indicated that lipid peroxidation occurred in the mouse liver. However, there was little effect of EGCG on GSH/GSSH in control and/or iron overload mice.

### 3.5. EGCG Addition Inhibits Iron Overload-Induced Ferroptosis by Elevating Antioxidant Capacity

**Figure 6:**
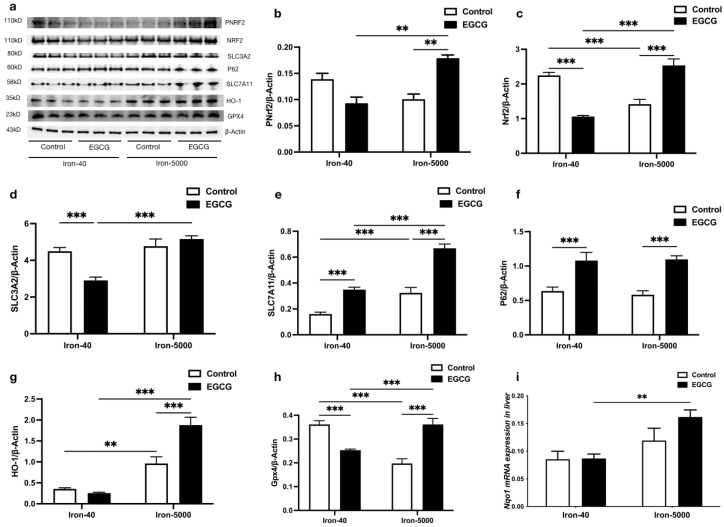
EGCG supplementation mitigates iron overload-induced oxidative damage by modulating antioxidant factors. (**a**) Western blot for the antioxidant-related protein expression (*n* = 3). (**b**–**h**) Quantification of pNRF2, NRF2, SLC3A2, SLC7A11, P62, HO-1, and GPX4 in the mouse livers of each group (*n* = 3). (**i**) mRNA expression of *Nqo1* (*n* = 10). The results of the two-way ANOVA are denoted by *p*-values, *p* < 0.01 (**), *p* < 0.001 (***).

Ferroptosis is a modality of cell death that is caused by iron-dependent peroxidation of lipids [3]. Most classic ferroptosis promoters are inhibitors of the antioxidant system. In order to further elucidate how ferroptosis causes lipid peroxidation and the antioxidant mechanism of EGCG, we measured the following antioxidant gene and protein expressions in iron overload mouse livers. From Figure 6, it is easy to see that iron overload significantly decreased the protein expression of NRF2, while EGCG addition rescued it, and the expression patterns of pNRF2 were consistent with it, which certified that EGCG invariably corrects the oxidative damage caused by iron overload (*p* < 0.01) (Figure 6a–c). Subsequently, we found that EGCG reduced SLC3A2 expression only in controls (Figure 6a,d) (*p* < 0.001), while hepatic SLC3A2 was insensitive to excess iron intake. In addition, high iron feed elevated the protein expression of SLC7A11 in the liver (*p* < 0.001), while having little effect on P62 (Figure 6a,e,f). Moreover, EGCG enhanced P62 and SLC7A11 protein expression under both normal and iron overload conditions (*p* < 0.001), which may have activated feedback to accumulate GSH and revealed the strong potential ability of EGCG as an antioxidant. Then, we measured the expression levels of NRF2′s downstream target protein HO-1, and the results showed that iron overload increased the HO-1 protein level (*p* < 0.01). EGCG has almost no effect on HO-1 in normal mouse livers, but it significantly increased HO-1 expression under iron overload conditions (*p* < 0.01) (Figure 6a,g). As a key part of the ferroptosis inhibiting system, the difference in the protein expression of GPX4 is particularly significant under different circumstances. Excessive iron caused this classic antioxidant’s expression to decrease, while this oxidative injury disappeared after EGCG administration (*p* < 0.001). Unexpectedly, adding EGCG suppressed GPX4 expression under normal conditions (Figure 6a,h). As a typical NRF2 target gene with the same role as HO-1, the *Nqo1* gene is consistent with HO-1 in terms of sensitivity to iron overload and the effect of EGCG. To sum up, these results demonstrated that EGCG inhibited ferroptosis via its potential antioxidant ability.

### 3.6. EGCG Addition Inhibits Iron Overload-Induced Ferroptosis by Altering Iron Metabolism

**Figure 7:**
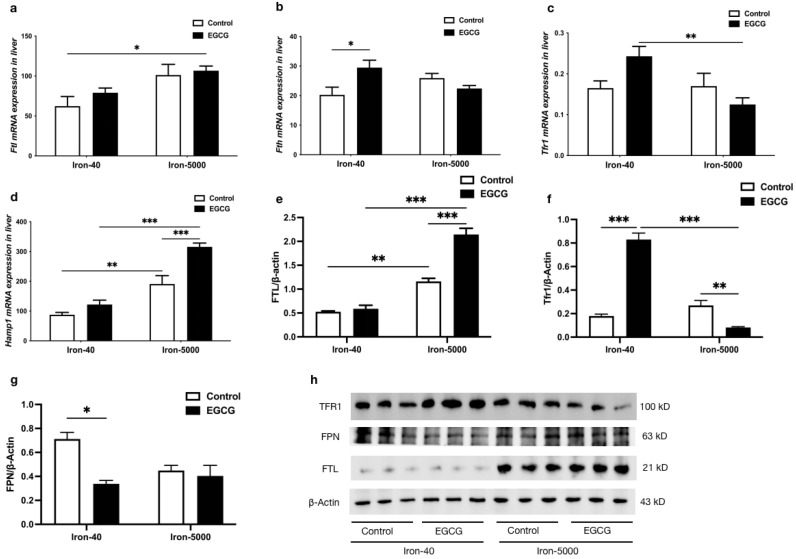
EGCG modulates iron metabolism factors to maintain iron homeostasis in mice. (**a**–**d**) *Ftl*, *Fth*, *Tfr1*, and *Hamp1* mRNA expression were tested by qPCR (*n* = 10). (**e**–**g**) Quantification of FTH/L, TFR1, and FPN in the liver of each group of mice (*n* = 3). (**h**) Western blot for FTH/L, TFR1, and FPN in the liver of each group of mice (*n* = 3). The results of the two-way ANOVA are denoted by *p*-values, *p* < 0.05 (*), *p* < 0.01 (**), *p* < 0.001 (***).

To determine the role of EGCG addition in iron overload-induced ferroptosis, iron metabolism-related gene and protein expressions were also tested. Figure 7 shows that the mRNA expression of *Ftl* and *Fth* in the liver were both upregulated in iron overload mice, as was the protein level of FTH/L (*p* < 0.01). Additionally, EGCG increased FTH/L expression levels in both normal and iron overload conditions (Figure 7a,b,e,h), which is beneficial for the increased storage of iron in mice. No significant trend of elevated mRNA and protein expression of TFR1 was induced by excessive iron intake, but it was significantly decreased by the addition of EGCG, while it caused a highly significant increase in TFR1 gene and protein expression in the control group (Figure 7c,f,h) (*p* < 0.01). Moreover, iron overload and EGCG both significantly increased the mRNA expression level of *Hamp1* (Figure 7d) (*p* < 0.01). Conversely, the protein expression of FPN decreased to various degrees due to the addition of iron or EGCG (Figure 7g,h).

## 4. Discussion

Iron is an indispensable trace metal in various physiological activities, such as respiration, oxygen transport, DNA synthesis, and energy generation [31]. If iron intake is overloaded or insufficient, it will lead to the interconversion of oxidative states, disturbing iron homeostasis and causing DNA damage, protein denaturation, and lipid peroxidation, collectively referred to as ferroptosis [32]. Iron deficiency develops into microcytic anemia in mice [33]. This is the most common type of anemia around the world [34], but excessive iron will induce ferroptosis and restrain erythropoiesis, also leading to anemia [35]. We found that iron overload causes body weight loss in mice and impairs hemoglobin production, leading to anemia. Furthermore, more free iron accumulates in the liver and spleen, and iron overload leads to abnormal AST/ALT ratios; an AST/ALT ratio over 2:1 has been implicated in liver damage [36,37]. However, the reason why the ALT and AST decreased under iron overloaded remains unclear. However, abnormally elevated liver MDA and reduced serum GSH/GSSG both indicate that iron overload caused oxidative damage in mice, consistent with our previous findings [29].

Excessive iron intake disrupts iron metabolism and produces significant changes in serum iron [38], which results in severe damage to red blood cells. Iron overload leading to reduced serum transferrin saturation (TF) has also been studied [39]. Anemia of inflammation is caused by the reduction in the lifespan of erythrocytes, impaired proliferation of red blood cells, and iron accumulation in the cells of the mononuclear phagocytic system via generation of proinflammatory cytokines, such as *IL-6*, causing hypoferremia [40]. Increased hepatic iron is also associated with liver inflammation [41]. In our study, iron overload significantly increased the mRNA or protein expression of *Ftl, Tfr1*, and *Hamp1*, in agreement with the results in other studies [42,43]. FTL/H is the storage ferritin, which can be degraded by lysosomes and increase the free iron content [44]. Moreover, iron homeostasis depends on the interaction of the iron hormone hepcidin with its receptor, iron exporter ferroportin (Fpn), and our experimental results also suggested the importance of the Hamp1-Fpn regulatory axis in the iron metabolic pathway [45]. On the other hand, excess iron may directly generate excessive ROS through the Fenton reaction, thereby increasing oxidative damage and ferroptosis [3]. Therefore, most classic ferroptosis promoters are inhibitors of the antioxidant system [46]. In the present study, Western blotting analysis showed that the decrease in P62-pNRF2/NRF2 is one of the main pathways of this ferroptosis event [11]. In addition, GPX4 and SLC7A11 function as classic negative regulators of ferroptosis and phospholipid hydroperoxidase by imposing restrictions on ROS production and cellular iron decrease. Obviously, iron overload directly increases SLC7A11 and SLC3A2 expression and inhibits GSH production, which in turn reduces the expression of downstream GPX4, enhances oxidative damage and lipid peroxidation, and promotes ferroptosis.

The mechanisms of action between EGCG and iron are complex and likely to be multifaceted. EGCG restores body weight in mice that consumed too much iron, a finding that has not been reported before. However, the weight loss of the control mice may have been due to the anti-obesogenic effects of EGCG [47] and the inflammation caused by excessive iron [48]. Whether EGCG upregulates or downregulates iron depends on the cell state and the dose of EGCG. Unexpectedly, the addition of EGCG recovered ferroptosis anemia in the iron overload mouse model, which was also a new finding for the health benefits of EGCG. However, these results were different from previous results showing that EGCG always abates hepatic iron by encouraging iron chelation therapy [49]. Moreover, EGCG achieved anti-inflammatory efficacy by inhibiting the expression of the proinflammatory factors *IL-1β* and *IL-16* [50,51,52], and reduces hepatotoxicity by decreasing MDA and the AST/ALT ratio in the mouse liver [53,54], suggesting that EGCG has beneficial pharmaceutical and medicinal properties [55].

EGCG’s antioxidant mechanisms are as follows. First, because of EGCG’s antioxidant properties, mitochondrial function is enhanced [56]. Additionally, EGCG reduces lipid infusion-mediated insulin resistance, which is connected to an increase in the expression of antioxidant enzymes such as superoxide dismutase (SOD) and glutathione peroxidase in vivo [57]. In our study, EGCG reduced high MDA and increased low serum GSH caused by iron overload. At the molecular biological level, EGCG induces the expression of GSH via the P62-NRF2 pathway, and NRF2 also directly regulates the activity of the HO-1 promoter. On the other pathway, SLC7A11 and SLC3A2 sustain the production of GSH, and more GSH elevates GPX4 expression downstream. In addition to antioxidants, EGCG also maintains iron homeostasis in mice by regulating iron metabolism. Furthermore, treatment of cells with iron chelators such as EGCG increases *Tfr* mRNA levels and protein expression [58]. The presence of EGCG chelators inhibits iron uptake by the TFR–Fe complex [59]. In the liver of iron-overloaded mice, EGCG reduces intracellular divalent iron ions and maintains iron ion homeostasis by reducing the expression of *Tfr1* and increasing that of *Ftl* and *Hamp1* genes. This can significantly reduce intracellular free iron content and ROS production, and even the Fenton reaction, inhibiting ferroptosis. EGCG also encourages cytotoxicity in anti-tumor activity in two ways: producing hydrogen peroxide with its pyrogallol moiety, or reducing Fe^3+^ to Fe^2+^, triggering the Fenton reaction, which produces more potent ROS such as hydroxyl radicals [60]. Thus, the bioavailability of EGCG is concentration-dependent.

Overall, in this study, we established mouse models of iron overload and EGCG by autonomous feeding and gavage to simulate daily diets, and determined the effects and the possible mechanisms of EGCG in regulating iron metabolism and alleviating oxidative damage caused by iron overload. We found that EGCG can prevent weight loss and treat anemia caused by iron overload in mice. Many studies have demonstrated how to improve the bioavailability of EGCG by structurally modifying it [61,62]. Under the conditions of the global pandemic of COVID-19, EGCG can suppress ACE2 (a cellular receptor for SARS-CoV-2) via activating Nrf2, inhibiting the main protease of SARS-CoV-2. In this way, EGCG may inhibit COVID-19 viral reproduction [63]. In view of the antioxidant effects of tea components such as EGCG, we clarified the potential health benefits of daily tea consumption and the need for more research on EGCG, which may become a novel and effective natural antagonist for ferroptosis-related diseases.

## 5. Conclusions

Our study revealed that iron overload can disrupt iron homeostasis and induce ferroptosis in mice, causing oxidative damage and anemia. EGCG is effective in alleviating oxidative damage caused by iron overload by inhibiting ferroptosis. These findings demonstrate the potential efficacy of EGCG in attenuating ferroptosis-related diseases.

## Data Availability

Not applicable.

## Supplementary Materials

The following supporting information can be downloaded at: https://www.mdpi.com/article/10.3390/nu15081993/s1.

## Abbreviations

RBC	red blood cell count
HGB	hemoglobin
HCT	hematocrit
MCV	mean corpuscular volume
MCH	mean corpuscular hemoglobin
MCHC	mean corpuscular hemoglobin concentration
TIBC	total iron binding capacity
UIBC	unsaturated iron binding capacity
TF	transferrin saturation
ALT	alanine transaminase
AST	aspartate transaminase
FAC	ferric citrate
ROS	reactive oxygen species
MDA	malondialdehyde
GSH/GSSG	glutathione
*IL-1β/IL-6*	interleukin
Nrf2/pNrf2	nuclear factor erythroid 2-related factor 2
SLC3A2	solute carrier family 3 member 2
SLC7A11	solute carrier family 7 member 11
P62/SQSTM1	sequestosome-1
HO-1	heme oxygenase 1
GPX4	glutathione peroxidase 4
FTH/L	ferritin heavy/light chain
TRF1	transferrin receptor 1
FPN	ferroportin
Hamp1	hepcidin1
