# Supplementary material for: Epigallocatechin-3-Gallate Alleviates Liver Oxidative Damage Caused by Iron Overload in Mice through Inhibiting Ferroptosis
Source: Nutrients. 2023 Apr 21;15(8):1993. doi: 10.3390/nu15081993 (PMC10145929; doi:10.3390/nu15081993)
Supplement: Supplementary file 1 [file nutrients-15-01993-s001.zip › nutrients-2215629-supplementary.pdf]

Supplementary 1

The in vitro experiment results showed that iron overload significantly reduced cell viability, while EGCG could recover it. As important indicators of ferroptosis, the levels of Fe^{2+}, ROS, and C11-BODIPY were significantly increased under iron overload, while EGCG significantly decreased the C11-BODIPY level under FAC treatment. In addition, we used RSL3 to induce ferroptosis and found that both 50 and 100 μM of EGCG could significantly reduce the C11-BODIPY level. In vitro experiments also proved that EGCG could alleviate iron overload and RSL3-induced ferroptosis.

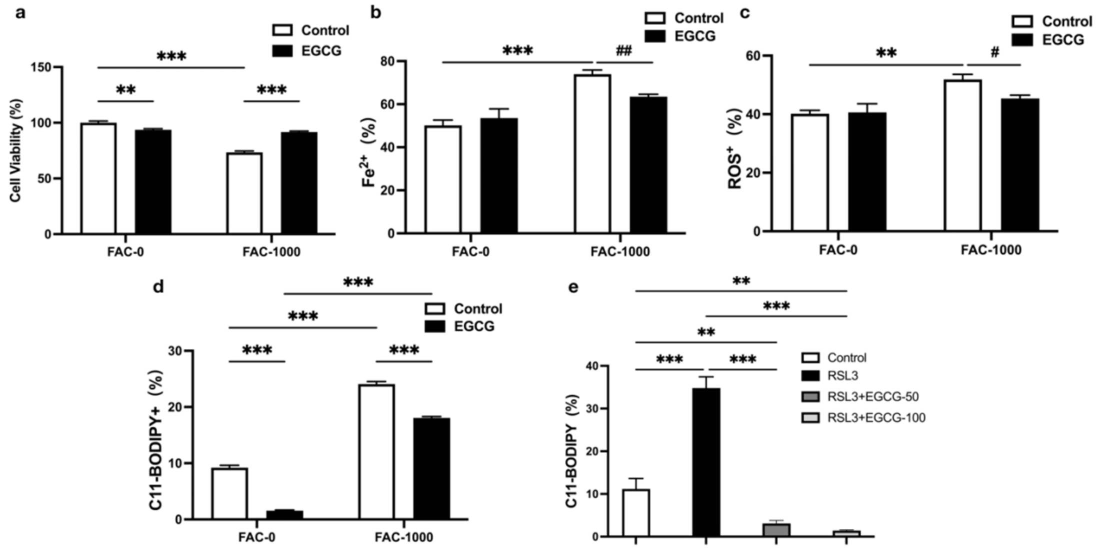

Figure S1. Iron overload induces ferroptosis, resulting in hepatocyte injury. (**a**–**d**) Cell viability, ferrous, ROS and C11-BODIPY level in the cells treated with FAC (n = 6) (Two-way ANOVA，$p < 0.05$ (*), $p < 0.01$ (**) and $p < 0.001$(***). *t*-test: $p < 0.05$ (#), $p < 0.01$ (##)). (**e**) C11-BODIPY level in cells with RSL3 (n = 3) (One-way ANOVA, $p < 0.05$ (*), $p < 0.01$ (**) and $p < 0.001$(***)).
